# Dopamine induces in vitro migration of synovial fibroblast from patients with rheumatoid arthritis

**DOI:** 10.1038/s41598-020-68836-z

**Published:** 2020-07-17

**Authors:** Lina van Nie, Laura Salinas-Tejedor, Nicole Dychus, Frank Fasbender, Marie-Lisa Hülser, Maurizio Cutolo, Stefan Rehart, Elena Neumann, Ulf Müller-Ladner, Silvia Capellino

**Affiliations:** 1 Department of Internal Medicine and Rheumatology, Justus-Liebig-University Gießen, Campus Kerckhoff-Klinik, Benekestr. 2-8, 61231 Bad Nauheim, Germany; 2 Department of Immunology, IfADo - Leibniz Research Centre for Working Environment and Human Factors, Ardeystrasse 67, 44139 Dortmund, Germany; 3 Research Laboratory and Academic Division of Clinical Rheumatology, Department of Internal Medicine, University of Genoa, Viale Benedetto XV 6, 16132 Genoa, Italy; 4 Department of Orthopedics and Trauma Surgery, Agaplesion Markus Hospital, Wilhelm-Epstein-Str. 2, 60431 Frankfurt, Germany

**Keywords:** Medical research, Autoimmunity

## Abstract

Preventing synovial fibroblast (SF) migration into the adjacent cartilage is a desirable therapeutic target in rheumatoid arthritis (RA). As previous studies demonstrated that RASF and SF from osteoarthritis (OA) patients express dopamine receptors (DR), aim of the present study was to investigate the impact of dopamine on mobility of fibroblasts from patients with chronic arthritides. Synovial tissue and fibroblasts were obtained from RA and OA patients. Immunohistochemistry was performed for all DR-subtypes in the invasion zone. Migration- and motility-assays were performed under DR-stimulation. Cytokines were evaluated using ELISA. Expression of DRs was evaluated by flow cytometry, and DR activation was measured by xCELLigence real-time analysis. All DRs were expressed in RA invasion zone. Migration and motility of RASF and OASF were increased after DR stimulation in patients ≤ 75 years old. Synovial fibroblasts from older RA patients (> 75 years old) expressed lower levels of D1-, D2- and D4-DR than patients ≤ 75 years old. DR activation was not altered in older patients. Our results suggest a possible involvement of dopamine on migration of fibroblasts from arthritis patients. Therefore, the synovial dopaminergic pathway might represent a potential therapeutic target to interfere with progressive joint damage in RA patients.

## Introduction

Rheumatoid arthritis (RA) is a chronic inflammatory disease characterized by synovial inflammation and progressive joint destruction, which leads to joint deformation and disability. Increasing evidences demonstrate that RA synovial fibroblasts (RASF) are among the key players in joint destruction. For instance, it was shown in a mouse model that activated RASF are able to transmigrate in other joints and are therefore responsible for spreading the disease to the majority of the joints^1^. The invasive phenotype of the activated fibroblasts is not dependent from inflammatory processes, as RASF are able to invade and destroy human cartilage and bone in the absence of immune cells^2^. This “aggressive” phenotype is particularly relevant in RA fibroblasts. Indeed, also SF from osteoarthritis (OA) patients are activated due to the presence of a damaged cartilage, but RASF are more aggressive compared to OASF^2,3^. Thus, preventing synovial fibroblasts (SF) from migrating into the adjacent cartilage is a desirable therapeutic target in RA in order to reduce joint destruction and disability.

Clinical evidences suggest a possible involvement of the dopaminergic pathway on RA^4^. Schizophrenia patients, undergoing pharmacological treatment with dopaminergic antagonists, have a low incidence of RA^5^, whereas Parkinson patients have a higher incidence of RA^6^. Moreover, RA patients often develop restless leg syndrome, a disorder involving the dopaminergic system^7^. Despite these clinical evidences, a possible direct role for dopamine in arthritis was suggested only within the last years in RA patients^8^ and in experimental arthritis^9,10^.

In the brain, dopamine is a neurotransmitter of the sympathetic nervous system, responsible for reward-motivated behavior. Dopamine is also involved in several signaling pathways outside of the central nervous system. For example, dopamine receptors (DRs) are expressed by immune cells and play a crucial role in modulating immune functions in the physiological situation but also during pathologic conditions^11–13^. Dopamine acts on five different DRs belonging to the G protein–coupled receptor family, which are grouped into 2 families: the D1-like DRs (D1DR and D5DR), which activate adenylate cyclase, and the D2-like DRs (D2DR, D3DR, and D4DR), which inhibit adenylate cyclase^14^.

In RA, dopamine is synthesized in inflamed synovial tissue^15^ and DRs are strongly expressed specifically by synovial fibroblasts, thus suggesting a functional role for dopamine in fibroblast activity in RA^8^. However, it was not yet demonstrated if the overexpression of DR in synovial fibroblasts from arthritic patients is linked to their aggressive phenotype. Therefore, the aim of this study was to investigate a potential involvement of dopamine in the aggressive phenotype of fibroblasts in RA patients.

## Results

### DRs are expressed at the invasion zone in RA and OA patients

**Figure 1:**
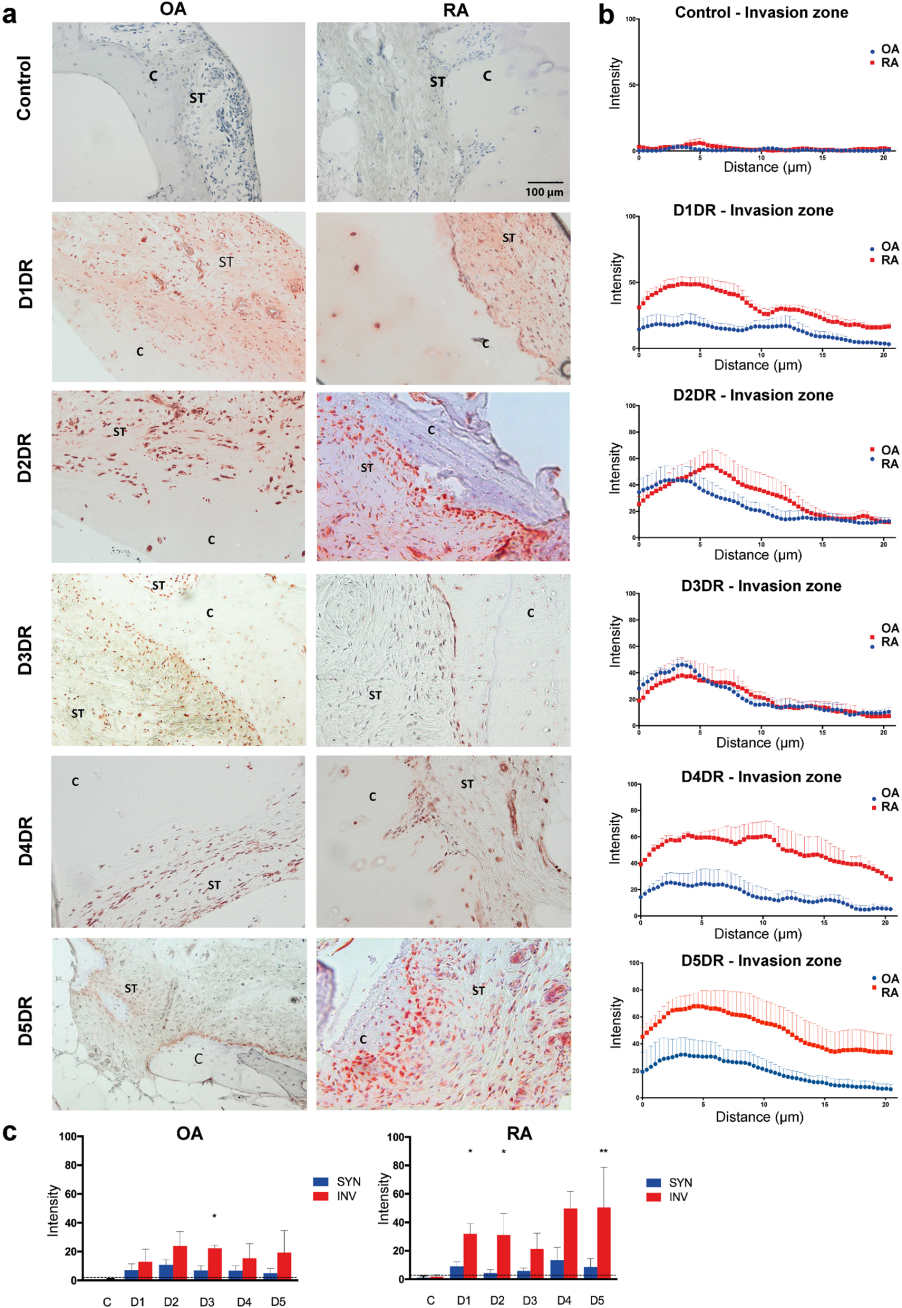
Immunohistochemical analysis of DR expression in OA and RA cartilage/synovium invasion zone. Dopamine receptors (D1DR-D5DR) were stained in paraffin-embedded tissue samples from osteoarthritis (OA) and rheumatoid arthritis (RA) patients. Red staining: DR. Purple staining: hematoxylin. *ST* synovial tissue, *C* cartilage, *B* bone. (**a)** Representative picture of at least 5 patients per each group. (**b)** Quantification of staining intensity as a function of distance from the cartilage. (**c**) Mean staining intensity in the invasion zone (INV, ≤ NV, μm from the cartilage or bone) compared to the other layers of the synovium (SYN). Mann Whitney test was used for comparison of groups. *P < 0.05; **P < 0.01.

Immunohistochemical analysis of DR expression revealed that all five DRs were expressed in the RA invasion zone (Fig. 1a, b). Particularly, the staining intensity of D1DR, D2DR and D5DR was stronger in the invasion zone than in the other layers of the synovial tissue in RA (Fig. 1c), and D3DR was significantly higher expressed in OA synovial tissue near the cartilage compared to the other layers of synovium (Fig. 1c). In general, DR intensity in the invasion zone was stronger in RA than in OA (Fig. 1c).

### Stimulation of the dopaminergic receptors leads to an increased synovial fibroblast migration in patients less than 75 years old

**Figure 2:**
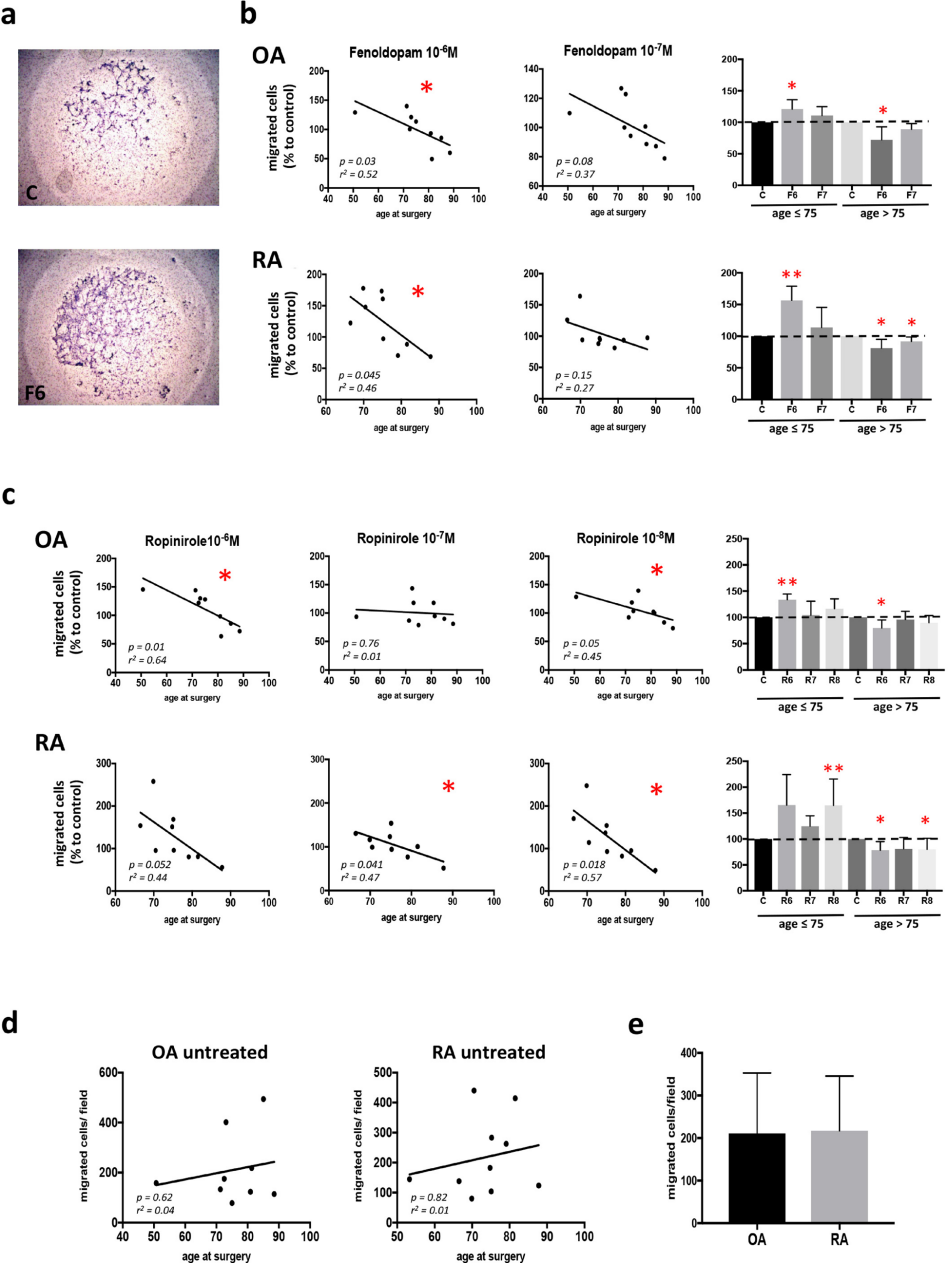
Effect of DR activation on SF migration. Migration of RASF (n = 9, with n = 5 younger than 75 years, and n = 4 older than 75 years) and OASF (n = 9, with n = 5 younger than 75 years, and n = 4 older than 75 years) was quantified by using a Boyden Chamber. Migrated cells were stained with hematoxylin and counted manually at the microscope. *C* control, *F* Fenoldopam, *R* Ropinirole; 6 = 10^–6^ M; 7 = 10^–7^ M; 8 = 10^–8^ M (**a**). Cell migration after stimulation of D1-like DRs with Fenoldopam at two different concentrations for 16 h (**b**). Cell migration after stimulation of D2-like DRs with different concentrations of Ropinirole for 16 h (**c**). Cell migration of unstimulated SF after 16 h of cell culture in the Boyden Chamber (**d**). Total migrated cells after 16 h of cell culture without dopaminergic stimulation in RA and OA (**e**). Results in (**b**) and (**c**) are shown as percentage to unstimulated control for each patient. Pearson correlation coefficients were used for statistical analysis of linear correlation, and Mann Whitney test was used for comparison of groups (histogram plots). *P < 0.05; **P < 0.01.

Stimulation of D1-like DRs with Fenoldopam led to a strong increase of SF migration (Fig. 2a) in younger patients (less than 75 years old), whereas cell migration was gradually inhibited with increasing age (Fig. 2b). Similar effects were observed after stimulation of D2-like DRs by Ropinirole (Fig. 2c). These age-dependent effects of dopamine were not due to an intrinsic lower migration ability of SF from older patients, as the migration capacity of untreated cells itself was not age-dependent (Fig. 2d). In a recently published paper from our group and conducted by using a very similar protocol, CD82 inhibition led to an approximately 50% increase of RASF migration after 16 h^16^. In the present study, we could observe a similar alteration in RASF migration in the younger patients. Therefore, the effect of dopaminergic stimulation observed in younger patients in this study is comparable to other stimuli used for this assay. Nevertheless, it is possible that not only dopaminergic agonists but also other substances are less efficient in inducing migration in older patients compared to younger ones. No differences were observed in the migratory capability between RA and OA patients (Fig. 2e).

### Cell motility is increased after dopamine receptor stimulation in patients less than 75 years old

**Figure 3:**
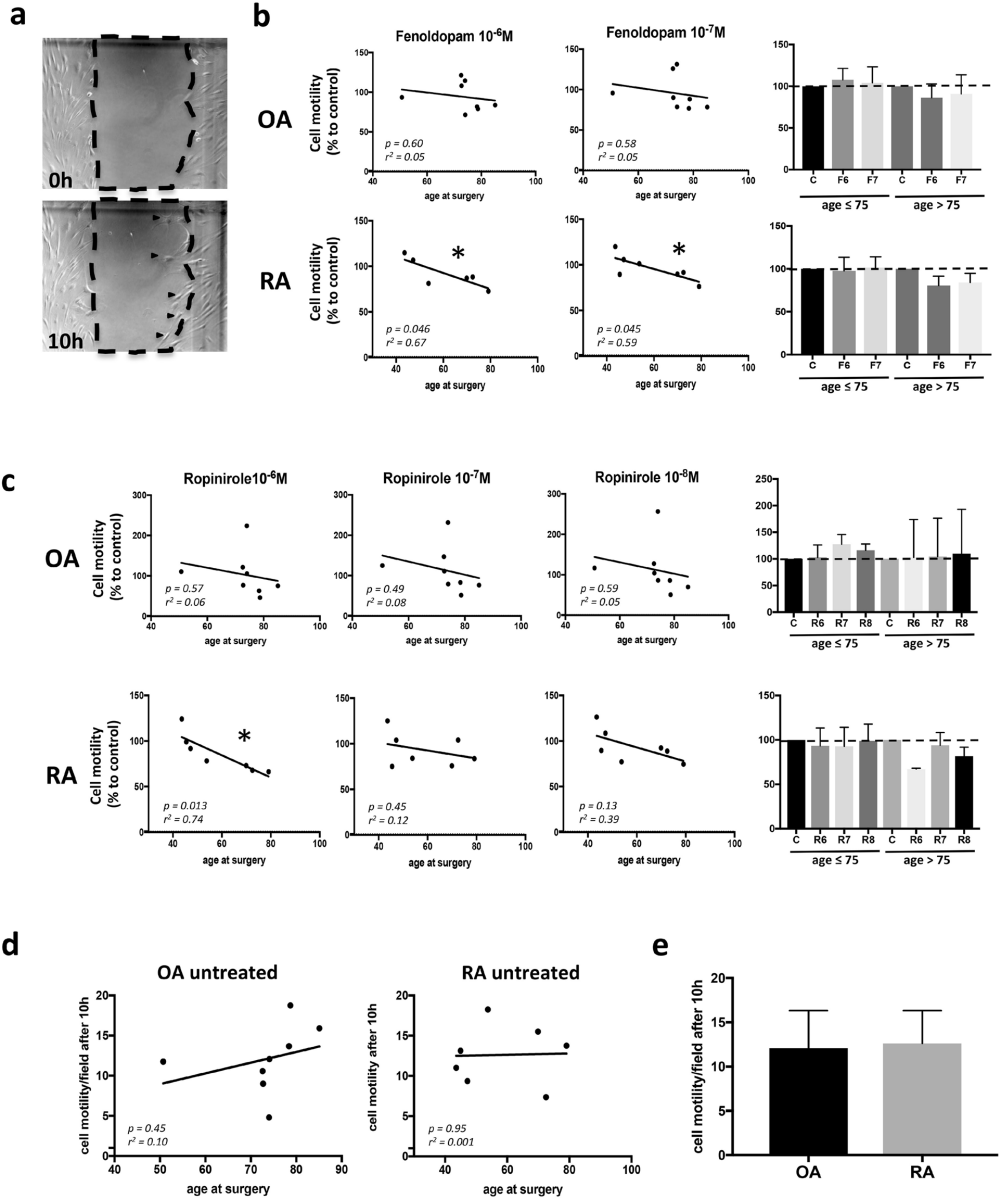
Effects of DR activation on cell motility. Cell motility of RASF (n = 7 with n = 4 younger than 75 years, and n = 3 older than 75 years ) and OASF (n = 8, with n = 4 younger than 75 years and n = 4 older than 75 years in OA) was quantified by using the scrape motility assay. Representative picture of cells moving into the scrape after 10 h of culture with normal medium (**a**). Cell motility after stimulation of D1-like receptors with Fenoldopam at two different concentrations for 10 h (**b**). Cell motility after stimulation of D2-like receptors with Ropinirole at three different concentrations for 10 h (**c**). Cell motility of unstimulated SF after 10 h (**d**). Total amount of cells that moved into the scrape after 10 h of cell culture without dopaminergic stimulation (**e**). Results in b and c are shown as percentage to unstimulated control for each patient. *C* control, *F* Fenoldopam, *R* Ropinirole; 6 = 10^–6^ M; 7 = 10^–7^ M; 8 = 10^–8^ M. Pearson correlation coefficients were used for statistical analysis of linear correlation, and Mann Whitney test was used for comparison of groups (histogram plots). *P < 0.05.

To confirm the previous results, we also performed the motility assay. As described above for cell migration, also cell motility of SF was influenced by dopamine (Fig. 3). D1-like stimulation with Fenoldopam for 10 h showed a significant correlation between motility and age in treated RA fibroblasts. No significant effects were detected in OA patients (Fig. 3b). However, due to the higher mean age of OA patient, it is possible that no correlation was observed due to the lack of data in the lower age range. Future experiments on a younger OA cohort will be required to confirm these results. D2-like stimulation with Ropinirole led to comparable effects (Fig. 3c). Similar results were also observed after 12 and 14 h (data not shown).

Similar to the results in the cell migration experiments, cell motility was not intrinsically correlated to the age of the patients (Fig. 3d), and no differences were observed in cell motility at baseline between RA and OA patients (Fig. 3e).

### D2-like DR stimulation has no strong effects on cytokine release

**Figure 4:**
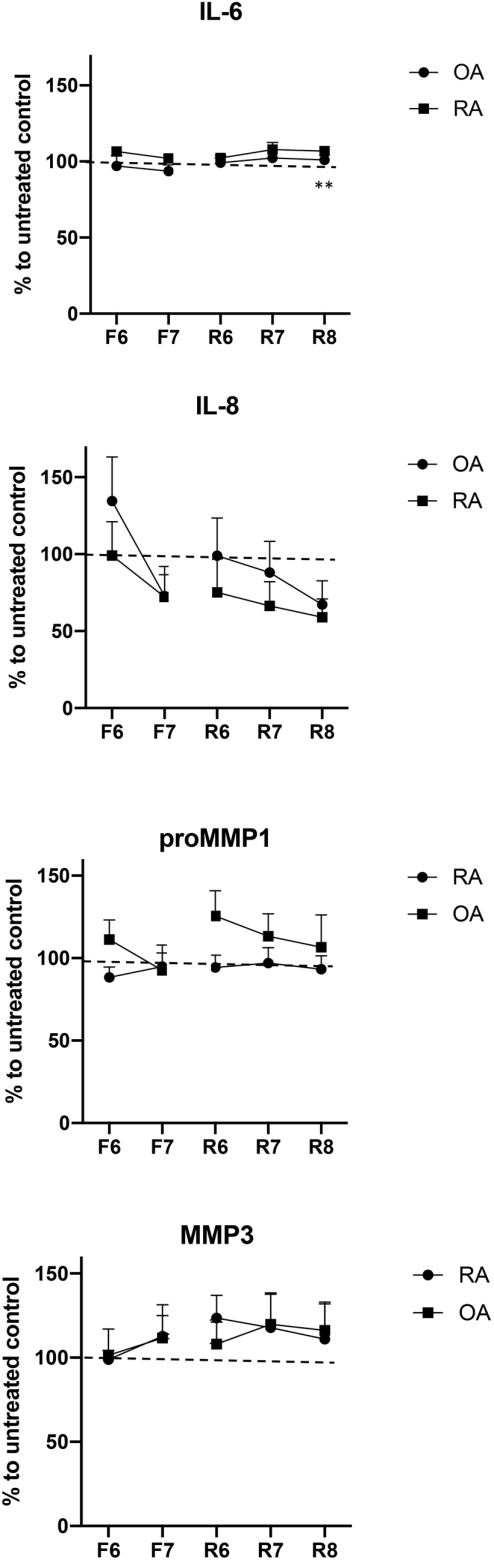
Cytokine release after DR stimulation. Quantification of IL-6, IL-8, proMMP-1 and MMP-3 released by RASF (n = 6–9) and OASF (n = 6–11) after 24 h of stimulation with Fenoldopam (F) or Ropinirole (R) at different concentrations (6 = 10^–6^ M; 7 = 10^–7^ M; 8 = 10^–8^ M). All results are shown as percentage (mean ± SEM) to untreated control. Wilcoxon matched-pairs signed rank test of raw data was used for comparison of treatments versus untreated control. **P < 0.005.

Stimulation of DR slightly increased IL-6 release in RA (Fig. 4), whereas it is doubtable that these small changes have any physiological relevance. IL-8 release tended to be lower in DR-treated RASF, but no significant differences to the untreated control were observed. The synthesis of matrix-degrading enzymes such as pro-MMP1 and MMP-3 was not influenced by the dopaminergic pathway (Fig. 4). This result suggests that dopamine’s main effects are on cell migration rather than on inflammation.

Baseline average levels of the cytokines were as follows: IL-8 in RA 40.16 ± 21 pg/ml and in OA 44 ± 35 pg/ml; IL-6 in RA 363.9 ± 221 pg/ml and in OA 308.6 ± 251 pg/ml; MMP-3 in RA 0.25 ± 0.13 ng/ml and in OA 0.34 ± 0.12; pro-MMP1 in RA 0.44 ± 0.2 ng/ml and in OA 0.40 ± 0.28 ng/ml (mean ± SD).

Cytokine release was not correlated to the age of the patient at time of surgery (data not shown).

### DR expression is reduced in older RA patients

**Figure 5:**
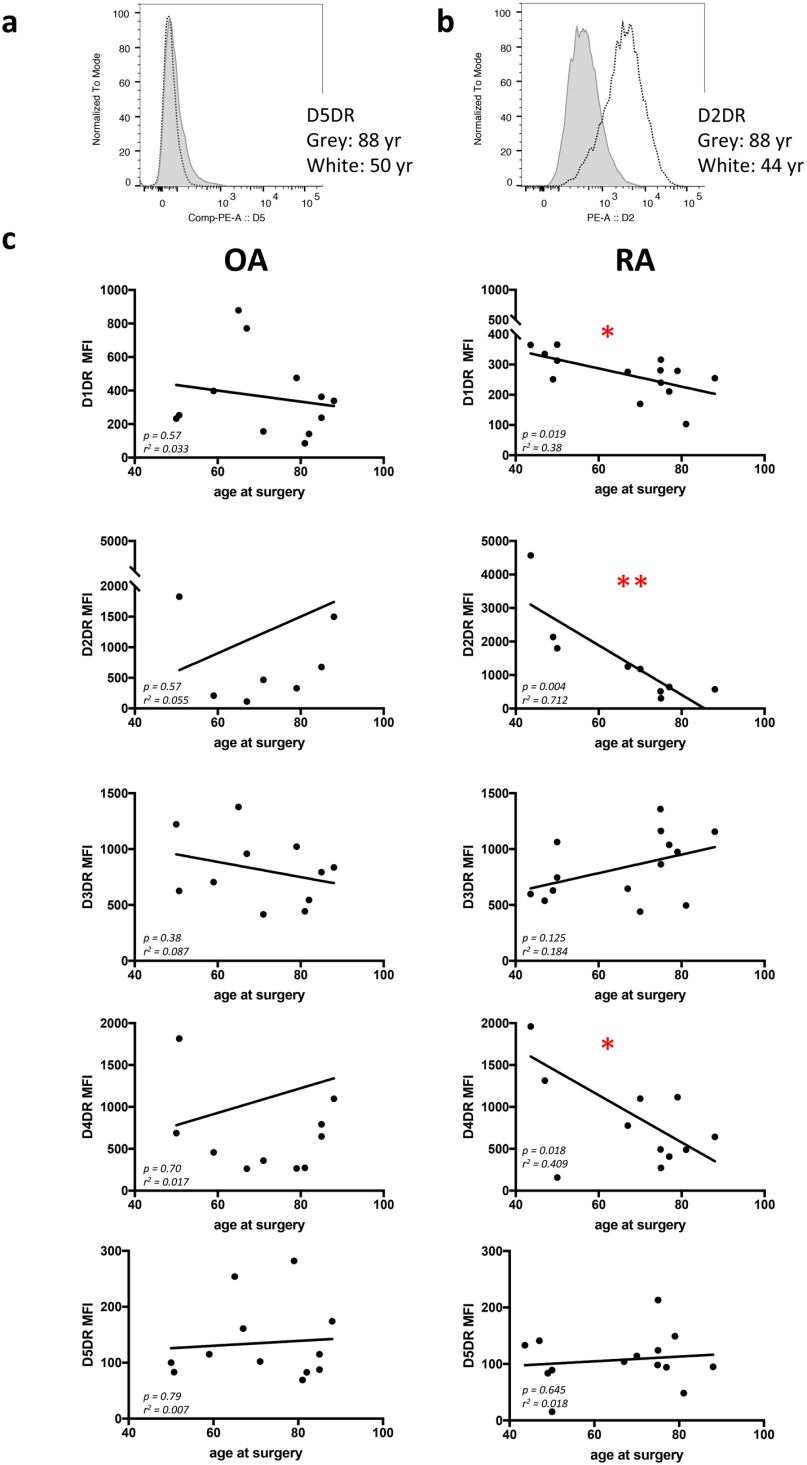
FACS analysis of DR expression. Representative pictures of D5DR FACS staining (**a**) and D2DR staining (**b**) in a younger (white) and older (grey) RA patient. DR expression in OA (n = 9–12) and RA (n = 11–15) patients in correlation to patients’ age at surgery (**c**). The y-axis indicates mean fluorescence intensity (MFI) measured for each receptor. Pearson correlation coefficients were used for statistical analysis of linear correlation. *P < 0.05; **P < 0.01.

FACS analysis of untreated RASF and OASF at the same culture passage used for the other experiments revealed that all DRs were expressed in cultured SF (Fig. 5a,b). Of interest, expression of D1DR, D2DR and D4DR was significantly lower in older RA patients, whereas no significant age-related differences were observed in OA patients (Fig. 5c).

Therefore, the reduced effects of dopaminergic stimulation on cell migration and motility in older RA patients could be at least partially due to a reduced DR expression.

### DR responsiveness is not altered in older patients

**Figure 6:**
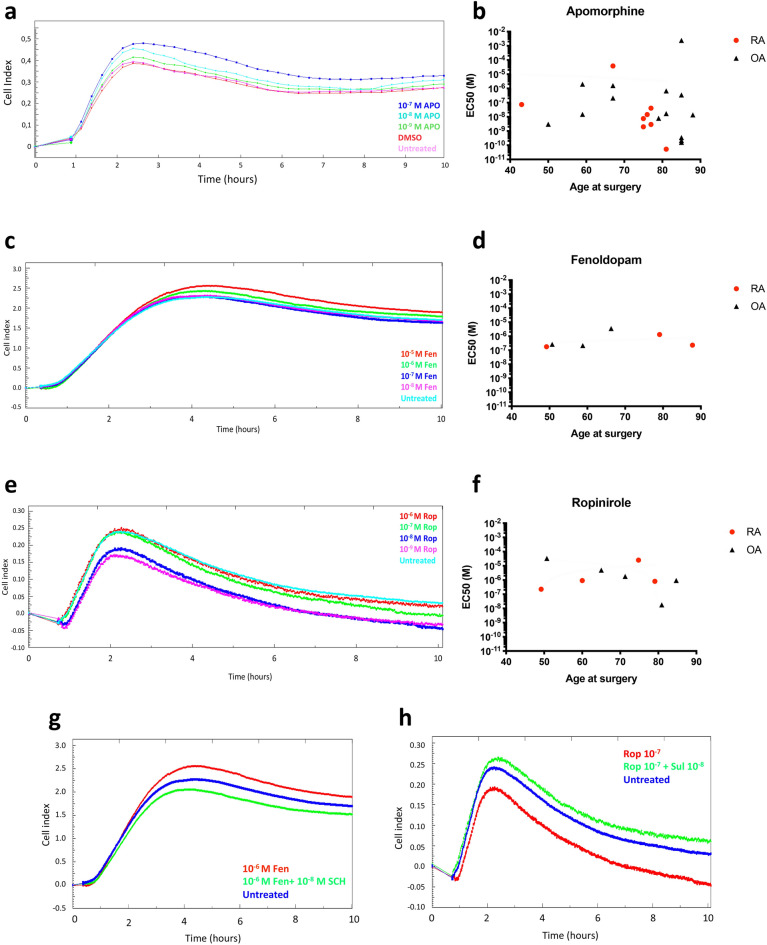
xCELLigence real-time cell analysis. RASF (total n = 12) and OASF (total n = 17) were treated with different concentrations of Apomorphine (APO) (**a**,**b**), a pan-specific DR agonist, Fenoldopam (fen) (**c**,**d**), the D1-like specific agonist, and Ropinirole (Rop) (**e**,**f**), the D2-like specific agonist. The cell index was evaluated for a total of 10 h. Representative pictures of cell index variations in correlation to the agonist concentrations in RA are shown in (**a**,**c**) and (**e**). EC50 values were calculated by using the RTCA software and then correlated to patients’ age at surgery (**b**,**d**,**f**). To prove if the used D1-like and D2-like agonists act via specific binding of DR, cells were co-treated with agonists and antagonists at different concentrations. The cell index was evaluated for a total of 10 h. Representative pictures of cell index variations (both from the same RA patient) are shown in (**g**) (D1-like DR) and (**h**) (D2-like DR). SCH = SCH39166, Sulp = S-(-)-Sulpiride.

In order to find out if DR responsiveness is reduced in older patients, we performed an RTCA analysis. Due to the similar results observed for D1-like and D2-like agonists in the experiments described above, a pan-specific DR agonist, Apomorphine, was used to define a dose–response curve for each patient. Although we observed a clear dose-dependent increase of the cell index in Apomorphine-treated SF when compared to untreated control or vehicle control (Fig. 6a), we did not find a correlation between age of the patient and the calculated EC50 (Fig. 6b). Also the treatment with the D1-like specific agonist Fenoldopam (Fig. 6c,d) and the specific D2-like agonist Ropinirole (Fig. 6e,f) showed a dose-dependent change in the cell index but no age-dependent effects. Taken together, these results suggest that DR are reduced in older patients but they are still properly responsive to the dopaminergic agonists.

We also performed experiments with the specific D1-like and D2-like agonists in combination with the respective antagonists (SCH39166 as D1-like antagonist and S-(-)-sulpiride as D2-like antagonist) at different concentrations. In all experiments, the co-treatment with specific antagonists reverted the effect of the agonist on the cell index, thus suggesting that the agonists specifically act via binding to the DR (Fig. 6g,h).

## Discussion

Joint destruction in RA can lead to severe functional and work disability^17^. In the last decades, several new therapies have been developed to slow down the progression of joint damage, but a treatment modality, which is able to stop synovial joint invasion and destruction of the adjacent cartilage and bone is still required^18^.

Our previous studies demonstrated a strong overexpression of DR specifically in synovial fibroblasts of RA patients, thus suggesting a role for dopamine on fibroblast function in RA pathology^8^, however, a possible influence of dopamine on joint invasion was not evaluated at that time. Of interest, also local dopamine synthesis was described in RA synovial cells, especially in fibroblasts, macrophages and B cells, suggesting that the dopaminergic pathways activated in RA synovial fibroblasts are regulated in an autocrine and paracrine way within the synovium^15^. Due to the fact that the studies were conducted with late-phase patients, it is not clear when during the inflammatory process the dopaminergic pathway started playing a crucial role in human. However, our previous data from the mouse model of arthritis suggest that this pathway could be activated quite early during the disease^19^. The results of the present study demonstrate that the dopaminergic receptors are strongly expressed in RA especially in the invasion zone in the joints (Fig. 1), and that the dopaminergic pathway is involved in the fibroblast migration in RA, underlining its potential role as non-canonical mechanism in joint inflammation and destruction.

Of interest, dopamine strongly induces cell migration and motility in younger patients, whereas cell motility and migration were progressively reduced in older patients. This age-dependent difference was not related to a reduced DR responsiveness in older patients, but could be due to an age-dependent switch of receptor activity, as previously demonstrated in dopaminergic^20^ and serotoninergic^21^ neurons. Another hypothesis is that a switch from G_αs_ to G_αi_ subunit of DRs occurs in synovial tissue during the course of disease, as previously described^22^, thus causing an opposite effect of DR stimulation in older patients with a longer disease history. Also, DR expression could be downregulated during the disease, as a result of long-lasting dopaminergic stimulation. This would explain why older patients, with longer disease duration, express less DR than younger patients. This hypothesis still needs to be confirmed by experimental data. Due to the fact that the majority of RA patients enrolled in this study are younger than 75, our results on older patients need to be confirmed in further studies. However, the majority of RA patients is younger than 75 years, therefore our results indicate that dopamine stimulation in most of the patients could lead to stronger fibroblast migration. Nevertheless, the gradual lost of dopamine effect on fibroblast migration in older patients should be addressed.

At present, the intracellular pathways activated by DR in RA synovial fibroblasts and responsible for cell migration are not clear. Indeed, cell migration is a highly complex process involving a broad variety of signal transduction depending on the matrix/adhesion molecule interactions and the microenvironment with altered chemokine, cytokine and growth factor concentrations within the tissue^23^. One of the possible intracellular pathways involved in cell migration is the cAMP pathway^23^, also involved in DR activation^14^. We therefore hypothesize that the cAMP pathway could be involved in DR-dependent fibroblast migration in RA. Future studies will be necessary to proof this hypothesis.

We also observed a reduction of DR expression on synovial fibroblasts from older RA patients. The reasons for the reduced DR expression specifically only in older RA patients still need to be elucidated. One possible explanation is that the expression of DR on synovial fibroblasts changes during disease progression and therapy, and older patients have in general a longer disease duration and therapy history compared to the younger patients. This hypothesis needs to be investigated as nothing is described so far about the expression of DR on synovial fibroblasts in the course of rheumatic disease and related to specific treatments. Nevertheless, a previous study on B cells from RA patients shows that D2-DR expression is altered during the course of disease and correlates with TNF levels and with therapy duration^24^ which supports the hypothesis that the dopaminergic phenotype can be altered also in RA fibroblasts during the disease and depending on the disease activity and treatment.

Activation of dopaminergic receptors tended to inhibit IL-8 release, but it did not reach a statistically significant reduction of IL-8 as observed in our previous findings^8^. Indeed, it should be pointed out that 50% of the here-analyzed RA patients were treated with biologics, whereas no patients with biologic treatment were included in our previous study. Therefore, a direct correlation of the results is not possible. It is plausible that treatment with biologics already dampens IL-8 levels, and that a further strong inhibition of IL-8 is thus no more reachable. Moreover, in the current study we used specific agonists for D1-like and D2-like DR for 24 h, to better distinguish between the two classes of DR, whereas in the previous study we cultured the cells with dopamine for 48 h. It is plausible that the stimulation of all DR at once and for longer time might lead to stronger effects on IL-8 secretion. In general, our findings are in line with the previous findings but suggest that dopamine’s main effects in long-treated RA patients are on cell migration rather than on inflammation.

Of note, no differences in cell migration and motility were observed between D1-like and D2-like DR stimulation. This could be due to a switch from G_αs_ to G_αi_ subunit, as previously described in RA^22^, or due to different intracellular pathways activated by DR in fibroblasts compared to neurons and other already described cell types (i.e. T-cells). The similar effects of D1-like and D2-like DR in RA fibroblast migration would simplify a possible use of the dopaminergic pathway as therapeutic target against joint destruction.

In conclusion, these findings demonstrate for the first time an involvement of the dopaminergic pathway in migration of synovial fibroblasts, supporting the therapeutic potential of the dopaminergic pathway in RA.

## Methods

### Tissue specimens and cell culture

31 patients with RA and 28 patients with OA, who underwent knee joint replacement surgery were included into the study (Table 1). As shown in Table 1, patient’s age at surgery as well as the gender distribution differ between the two groups. These differences reflect the demographic features of these two cohorts, as OA has normally a later onset compared to RA, and RA affects more women than men^25,26^. All patients were informed about the purpose of the study and gave written consent. The study was approved by the Ethics Committee of the Justus-Liebig-University of Giessen. All investigations conformed to the principles outlined in the Declaration of Helsinki.

**Table 1:** Characteristics of the patients included in the study.

Characteristic	Osteoarthritis (n = 28)	Rheumatoid arthritis (n = 31)
Age, mean (years)	74.4	63.3
Age range (years)	58.8–88.5	27.4–87.8
Disease duration, mean (years)	N.D	16.4
Disease duration range (years)	N.D	2–46
Men/women (%)	9/19 (32/68)	3/28 (10/90)
Medication^a^		
Glucocorticoids (%)	N.D	69
DMARDs (%)	N.A	52
Biologicals (%)	N.A	50

^a^Data about medication were not available for all patients. The percentage shown in the table is referred to the RA patients with complete data information (n = 26).

Tissue preparation for the histological analysis was performed as previously described^27^. In brief, synovial tissue specimens of the cartilage-synovium transition zone (each piece around 2–3 mm^3^) were fixed in 3.7% formaldehyde and then paraffin-embedded. 5 μM thick slices were used for histological analysis.

For in vitro cell culture, synovial fibroblasts were isolated by enzymatic digestion as previously described^2^, and cultured for 4 passages in DMEM medium added with 10% FCS, 1% HEPES, 100 U/ml of penicillin and 0.1 μg/ml streptomycin. All experiments were performed between passage 4 and 5 of cell culture^28^.

### Immunohistochemistry of DRs

Histological analysis of dopaminergic receptors (DRs) was performed on paraffin embedded tissue samples. After de-paraffinization and rehydration, endogenous peroxidases were blocked by incubation with 3% H_2_O_2_, followed by antigen retrieval. This was achieved by incubation at 92 °C in citrate buffer or by enzymatic digestion with proteinase K, depending on the receptor subtype. Non-specific binding sites were blocked with phosphate-buffered saline (PBS) containing 10% fetal calf serum, 10% chicken serum and 10% albumin. Samples were then incubated overnight at 4 °C with the primary antibody (mouse anti-hD1DR: Santa Cruz, cat. nr. sc-3360; rabbit anti-hD2DR: Acris, cat. nr. TA316680; mouse anti-hD3DR: BioLegend, cat. nr. 827501; rabbit anti-hD4DR: OriGene, cat. Nr. TA321201; mouse anti-hD5DR: Santa Cruz, cat. nr. sc-376088) and then counterstained by using Histofine (Nichirei Biosciences, cat. Nr. 414152F), as indicated by the manufacturer. Control staining with the respective isotype controls (D1DR: santa cruz, cat. nr. sc3879; D2DR and D4DR: DAKO, cat. nr. DAK-X090302-8; D3DR: Biolgend, cat. nr. 400201; D5DR: santa cruz, cat. nr. sc-3877) and with the secondary antibody alone (Histofine, Nichirei Biosciences, cat. Nr. 414152F) were carried out in parallel and showed no positive staining.

Dopamine receptor expression was quantified in the invasion zone and synovial tissue of OA and RA patients with ImageJ software. Invasion zone was defined as the area comprised between the cartilage or bone surface (x = 0) and 20 μm inside the synovial tissue. Four different measurements were evaluated per region and patient, and three to four patients were evaluated per group. Dopamine receptor expression intensity was represented as a function of distance in the invasion zone. Additionally, mean intensity value for each dopamine receptor was compared between both regions.

### Boyden migration assay

Migration of RASF (n = 11) and OASF (n = 9) was quantified by using a Boyden Chamber (Neuroprobe) (Meier et al. 2012). SF were exposed to culture medium containing 2% FCS for 6 h prior to the experiment. After collecting and counting the SF, 50 µl of culture medium containing 2% FCS and 30.000 cells were filled into the upper chamber. The lower chamber contained 30 µl culture medium with 10% FCS. Apart from wells for negative control, both upper and bottom wells also contained the specific receptor agonist, respectively (Fenoldopam: D1-like agonist, 10^–6^ M, 10^–7^ M; Ropinirole: D2-like agonist, 10^–6^ M to 10^–8^ M), solved in sterile water and then diluted in culture medium. The selected dopaminergic agonists are already approved as medication. Fenoldopam is a selective D1-like agonist used as an antihypertensive agent and Ropinirole is a D2-like selective agonist used to treat Parkinson's disease and restless legs syndrome. Both substances were from Tocris Bioscience. The concentrations used are based on the binding affinity of each substance, as described by the company and in the literature. Both chambers were separated by a membrane with 8 µm pores. After 16 h of incubation at 37 °C, migrated cells were fixed and stained with hematoxylin. Three fields per well were photographed (10 ×) and the migrated cells per field were counted. The results were presented as the difference of migration compared to the unstimulated control in percentage.

### Scrape motility assay

For the motility assay, RASF and OASF from 7 patients per group were observed (Meier et al., 2012). Cells were incubated in 48 wells until they reached 90–100% of confluence. Subsequently, a scrape was scratched in the middle of each well by using a 100 µl pipet tip. Cells were incubated with complete culture medium and with or without specific DR agonists (as indicated above) for 16 h. Photos of the whole scrape (10 × augmentation, phase contrast microscope) were taken directly after scraping and after 10, 12, 14 and 16 h and migrated cells were counted at the respective time points. Difference in cell motility under specific stimulation was expressed in difference to the unstimulated control at the same time point, expressed in percentage.

### Cytokine quantification

RASF (n = 11–15) and OASF (n = 9–12) were stimulated 24 h with Fenoldopam and Ropinirole as described above. Cell culture supernatants were then collected and stored at − 20 °C until use.

Pro-MMP1, MMP-3 and IL-6 release was determined by using commercially available ELISA kits (R&D, Quantikine ELISA). IL-8 release was evaluated by using a commercially available ELISA kit from BioLegend. The assays were performed as indicated by the manufacturers. Each samples was evaluated in triplicates and results are presented as mean ± SD.

### FACS staining of DRs

Untreated RASF (n = 15–11) and OASF (n = 13–8) from one confluent 12 well plate were used for the staining. Cells were gently detached by using an enzyme-free cell dissociation buffer (Life Technologies, cat nr. 13151–014) and then incubated with conjugated antibodies against D1DR, D3DR or D5DR (rabbit anti-hD1DR, FITC conjugated: Antibodies online, cat nr. ABIN2173733; rabbit anti-hD3DR, Cy5 conjugated: Bioss, cat nr. BSS-BS-1743R; rabbit anti-hD5DR PE conjugated: Novus Biologicals cat nr. FAB82861P).

D2DR and D4DR were stained with antibodies targeting cytoplasmatic domains. For the intracellular staining, SF were fixed with PBS + 2% formaldehyde, then permeabilized with a specific permeabilizing solution (BD Biosciences, cat nr. 340973) prior incubation with the specific primary antibody (rabbit anti-hD2DR: Biozol, cat nr. LS-A1405; rabbit anti-hD4DR: Biorbyt, cat nr. BYT-ORB39453). After washing, cells were incubated with the specific secondary antibody (donkey anti-rabbit PE, Biolegend, cat nr. 406421).

Cells were analyzed using the FACSDiva™ Software on a LSRFortessa™ cell analyzer (BD Biosciences). Mean fluorescence intensity of the gated population (see additional figure 1) was evaluated and used for further analysis. As controls, fluorescence-minus-one (FMO) staining was performed^29^ and showed no unspecific staining (see additional figure 2).

### xCELLigence real-time cell analysis

Dopamine receptors, like all G-protein coupled receptors (GPCRs) upon activation lead to signaling involved in changes in at least one of the following: cell size/shape, cell attachment, and cell proliferation. All these effects are analyzed by xCELLigence real time cell analysis (RTCA)^30–32^. Thus, RTCA is a suitable method for investigating DR-activation in synovial fibroblasts.

Synovial fibroblasts from RA (n = 12) and OA (n = 17) were resuspended in cell culture medium containing four different concentrations (10^–5^ M to 10^–8^ M) of Fenoldopam (D1-like agonist) or Ropinirole (D2-like agonist), alone or in combination with the respective antagonists (SCH39166 for D1-like and S-(-)-sulpiride for D2-like) at the concentration of 10^–8^ M. We also treated the cells with different concentrations (10^–7^ M to 10^–10^ M) of Apomorphine, a pan-specific dopaminergic agonist, to mimic dopaminergic effects. For each condition, cells were seeded in triplicates in E-plates of xCELLigence RTCA DP system (ACEA Biosciences Inc.). As control, cells without agonists were analyzed. The E-plates containing the cells were allowed to incubate at room temperature for 30 min before being placed on the device station in the incubator for continuous recording of impedance as reflected by cell index (CI). Cell Index was recorded every minute (for Apomorphine: every 5 min). RTCA software was used to calculate EC50 values.

### Statistical analysis

Two groups were compared by the non-parametric Mann–Whitney U test. Wilcoxon matched-pairs signed rank test was used for comparison of cytokine levels in DR-treatments versus untreated control. For linear correlation, the Pearson correlation coefficient was evaluated. GraphPad Prism 7 was used for the statistical data analysis and graphic design.

### Ethical approval and consent to participate

All patients were informed about the purpose of the study and gave written consent. The study was approved by the Ethics Committee of the Justus-Liebig-University of Giessen.

## Supplementary information

Supplementary file1 (PDF 427 kb)

Supplementary file2 (PDF 286 kb)

## Data Availability

All relevant data supporting our findings are included in this published article and its supplementary information files.

## Abbreviations

OA: Osteoarthritis
RA: Rheumatoid arthritis
SF: Synovial fibroblasts
DR: Dopaminergic receptor
RTCA: Real time cell analysis
FACS: Fluorescent activated cell sorting (flow cytometry)
