# Supplementary material for: Dopamine induces in vitro migration of synovial fibroblast from patients with rheumatoid arthritis
Source: Sci Rep. 2020 Jul 17;10:11928. doi: 10.1038/s41598-020-68836-z (PMC7368011; doi:10.1038/s41598-020-68836-z)
Supplement: Supplementary file 1 — Supplementary file1 (PDF 427 kb) [file 41598_2020_68836_MOESM1_ESM.pdf]

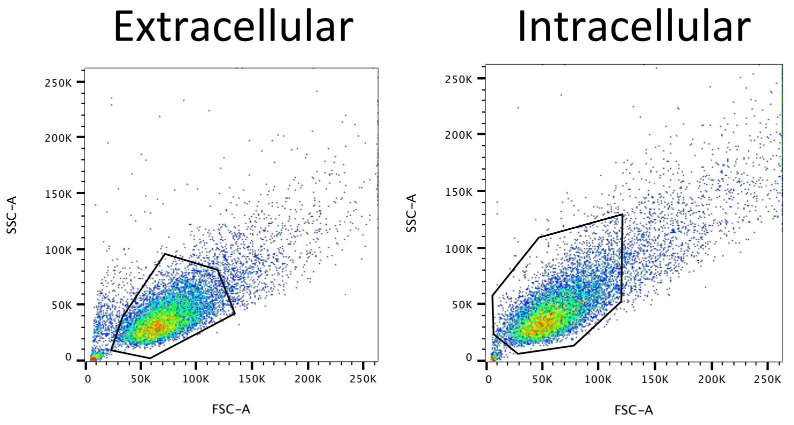

Figure S1: FACS gating strategy.
Gating strategy for extracellular stained fresh cells (left panel) and intracellular stained fixed cells (right panel).
