# Supplementary material for: Dopamine induces in vitro migration of synovial fibroblast from patients with rheumatoid arthritis
Source: Sci Rep. 2020 Jul 17;10:11928. doi: 10.1038/s41598-020-68836-z (PMC7368011; doi:10.1038/s41598-020-68836-z)
Supplement: Supplementary file 2 — Supplementary file2 (PDF 286 kb) [file 41598_2020_68836_MOESM2_ESM.pdf]

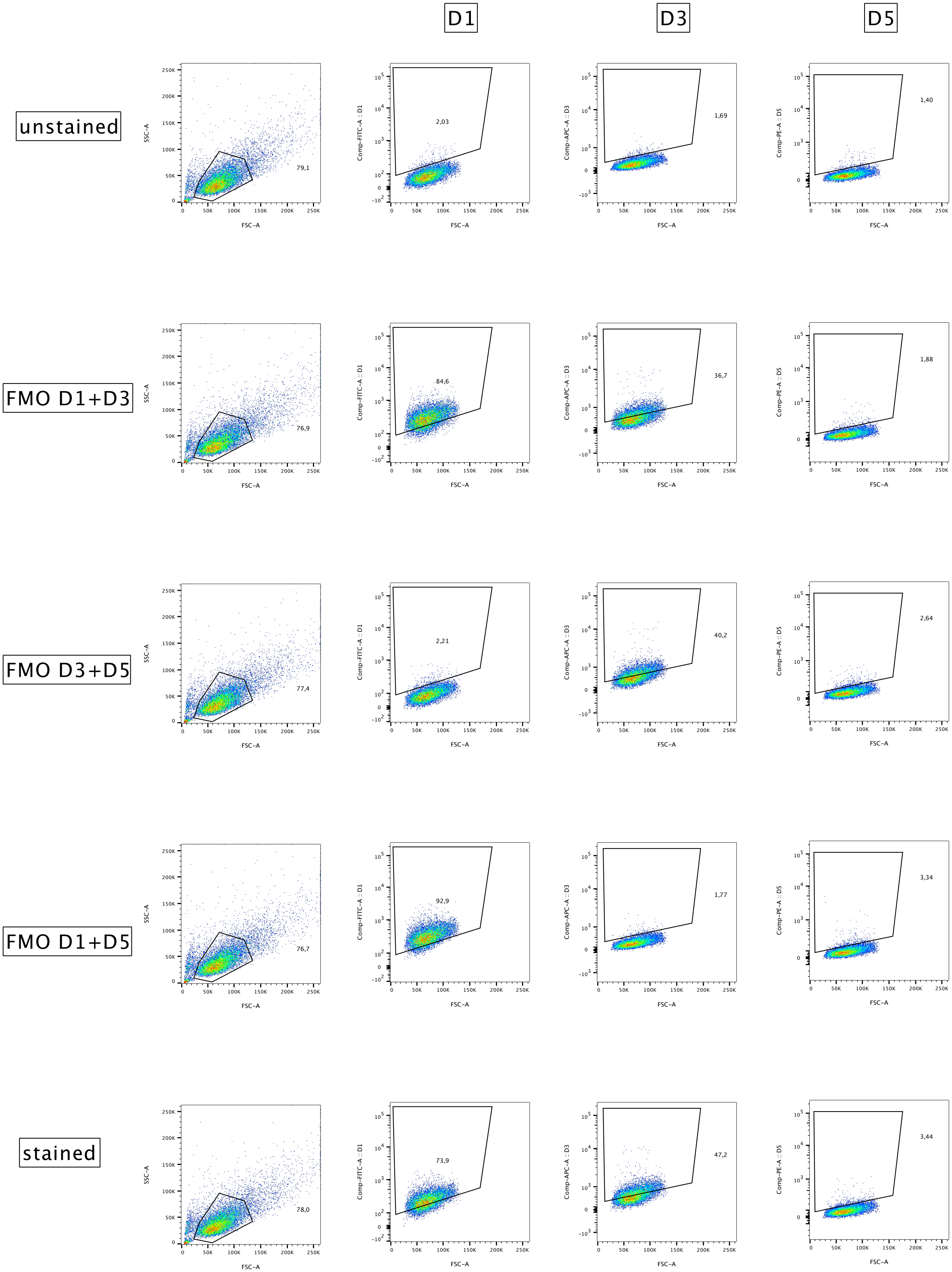

Figure S2: FACS negative controls.

Negative controls of the FACS staining are shown, as well as the gated population and the % of positive cells. In the upper row, unstained cells are displayed. In the lower row, stained cells for all markers are shown. The other rows present the FMO (fluorescence minus one) control for each marker, as displayed in the picture. D1 = dopamine receptor 1, D3 = dopamine receptor 3, D5 = dopamine receptor 5.
